# Editorial to “Can lead damage be ruled out using defibrillation threshold testing in patients with very high‐impedance shock leads?”

**DOI:** 10.1002/joa3.70040

**Published:** 2025-03-12

**Authors:** Taro Temma, Toshihisa Anzai

**Affiliations:** ^1^ Department of Cardiovascular Medicine, Faculty of Medicine and Graduate School of Medicine Hokkaido University Sapporo Japan

Defibrillation threshold (DFT) testing has long been debated in the field of implantable cardioverter defibrillator (ICD) management, with contemporary practice leaning towards its selective rather than routine use. The study by Narita et al.^1^ presents a compelling case where DFT testing played a pivotal role in evaluating the function of an ICD lead with very high shock impedance, ultimately guiding clinical decision‐making. Their findings provide valuable insights into the evolving role of impedance monitoring and highlight the limitations of low‐voltage subthreshold measurement (LVSM) in assessing true shock impedance (TSI).

The report describes a case in which an Endotak Reliance 0296 lead exhibited a progressive increase in shock impedance over 11 years, eventually surpassing 200 Ω. This raised concerns about potential lead dysfunction, necessitating a clinical approach to determine the safety, and efficacy of continued use. The authors convincingly demonstrate that despite the alarmingly high impedance recorded by LVSM, the lead remained functional, as confirmed by successful DFT testing with a true shock impedance of 103 Ω.

The discrepancy between LVSM and TSI is a critical finding. LVSM has been widely adopted for its non‐invasive, pain‐free nature, but, as shown in this case, it may not always provide an accurate reflection of true lead function. The authors postulate that lead encapsulation and environmental stress cracking may have contributed to the impedance increase. These factors, along with the known risk of calcification in GORE‐expanded polytetrafluoroethylene (ePTFE)‐coated coils, raise important considerations for long‐term lead surveillance.

DFT testing has been largely deemphasized in recent years due to concerns about procedural risks and limited impact on patient outcomes in standard ICD implants.^2^ However, this case underscores its utility in specific clinical scenarios. In the presence of suspected lead dysfunction, particularly with high‐voltage impedance concerns, DFT testing can provide a definitive functional assessment of lead replacement and decrease the LVSM to the normal range, making it easy to detect future lead fractures using LVSM. Furthermore, prior study suggests that a commanded low‐energy impedance test (0.1 Joule) is a safer and more reliable method for identifying and verifying potential open shock line conditions compared to high‐energy shock testing.^3^ This raises the possibility that a combined approach using both low‐ and high‐energy shocks could enhance the assessment of lead function in such cases. By incorporating both strategies, clinicians may improve diagnostic accuracy and ensure the long‐term functionality of ICD leads while minimizing unnecessary interventions. The study by Narita et al. presents a well‐documented case highlighting the complexities of ICD lead impedance monitoring. Their findings support the selective use of DFT testing as a valuable tool in assessing lead function when impedance values exceed conventional thresholds. This report serves as an important reminder that while non‐invasive impedance assessments are useful, they may not always provide a complete picture of lead integrity. In an era where device longevity and patient safety are paramount, careful interpretation of impedance trends, alongside judicious use of DFT testing, will remain crucial in guiding optimal ICD lead management. Ongoing research and improved lead surveillance strategies are needed to refine clinical decision‐making and optimize outcomes for ICD patients.

## FUNDING INFORMATION

Dr. Anzai (TA) reports grants from Abbott Medical Japan LLC, Japan Lifeline Co., Ltd., and Boston Scientific Co., Ltd., and scholarship funds from Biotronik Japan Co., Ltd., Medtronic Japan Co., Ltd., Win International Co., Ltd., Medical System Network Co., Ltd., and Hokuyaku Takeyama Holdings, Inc.

## CONFLICT OF INTEREST STATEMENT

Authors declare no conflict of interest for this article.

## References

[1] Narita M , Ikeda Y , Mori H , Kato R , Matsumoto K . Can lead damage be ruled out using defibrillation threshold testing in patients with very high‐impedance shock leads? J Arrhythm. 2025;41(1):e70014. 39950142 10.1002/joa3.70014 PMC11821546

[2] Phan K , Ha H , Kabunga P , Kilborn MJ , Toal E , Sy RW . Systematic review of defibrillation threshold testing at DE novo implantation. Circ Arrhythm Electrophysiol. 2016;9:e003357. 27030699 10.1161/CIRCEP.115.003357

[3] Kadosaka T , Watanabe M , Nakao M , Koya T , Temma T , Anzai T . Normalization of increasing shocking coil impedance with full output synchronized shock. J Cardiovasc Electrophysiol. 2024;35:2251–2253. 39169529 10.1111/jce.16416

